# Does Sleep Position Influence Sleep-Disordered Breathing in Infants With Cleft Palate: A Feasibility Study?

**DOI:** 10.1177/10556656211003459

**Published:** 2021-04-01

**Authors:** Clare S. Murray, Tanya Walsh, Trisha Bannister, Aleksandra Metryka, Karen Davies, Yin Ling Lin, Paula Williamson, Peter Callery, Kevin O’Brien, William Shaw, Iain Bruce, On behalf of the SLUMBRS Study Advisory Group (SAG) and Respiratory Group

**Affiliations:** 1 Division of Infection, Immunity and Respiratory Medicine, School of Biological Sciences, University of Manchester, Manchester, UK; 2 Royal Manchester Children’s Hospital, Manchester University Hospitals NHS Foundation Trust, Manchester Academic Health Science Centre, Manchester, UK; 3 Division of Dentistry, School of Medical Sciences, University of Manchester, Manchester, UK; 4 Cleft and Craniofacial Clinical Research Centre, Division of Dentistry, University of Manchester, Manchester, UK; 5 Division of Nursing, Midwifery and Social Work, University of Manchester, Manchester, UK; 6 Clinical Trials Research Centre, Institute of Translational Medicine, University of Liverpool, Liverpool, UK; 7 Manchester Clinical Trials Unit, School of Health Sciences, University of Manchester, Manchester, UK

**Keywords:** cleft palate, sleep-disordered breathing, oxygen saturation, infant sleep position

## Abstract

**Objective:**

Cleft palate (CP) can affect breathing, leading to sleep-disordered breathing (SDB). Sleep position can affect SDB, but the optimum sleep position for infants with CP is unknown. We aimed to determine the design of a pragmatic study to investigate the effect of the 2 routinely advised sleep positions in infants with CP on oxygen saturations.

**Design:**

A multicentered observational cohort.

**Setting:**

Four UK-based cleft centers, 2 advising supine- and 2 side-lying sleep positions for infants with CP.

**Participants:**

Infants with isolated CP born July 1, 2015, and December 31, 2016. Of 48 eligible infants, 30 consented (17 side-lying; 13 supine).

**Interventions:**

Oxygen saturation (SpO_2_) and end-tidal carbon dioxide (ETCO_2_) home monitoring at age 1 and 3 months. Qualitative interviews of parents.

**Outcome Measures:**

Willingness to participate, recruitment, retention, and acceptability/success (>90 minutes recording) of SpO_2_ and ETCO_2_ monitoring.

**Results:**

SpO_2_ recordings were obtained during 50 sleep sessions on 24 babies (13 side-lying) at 1 month (34 sessions >90 minutes) and 50 sessions on 19 babies (10 side-lying) at 3 months (27 sessions >90 minutes). The ETCO_2_ monitoring was only achieved in 12 sessions at 1 month and 6 at 3 months; only 1 was >90 minutes long. The ETCO_2_ monitoring was reported by the majority as unacceptable. Parents consistently reported the topic of sleep position in CP to be of importance.

**Conclusions:**

This study has demonstrated that it is feasible to perform domiciliary oxygen saturation studies in a research setting and has suggested that there may be a difference in the effects of sleep position that requires further investigation. We propose a study with randomization is indicated, comparing side-lying with supine-lying sleep position, representing an important step toward better understanding of SDB in infants with CP.

## Introduction

Cleft lip and palate is a common birth defect (1/700 births) of which approximately 44% have an isolated cleft palate (CP; 1/1600 births, National congenital anomaly and rare disease Registration service, 2016). Cleft palate results in a disruption to the function of the face and upper airway structures altering the efficiency of breathing. Upper airway obstruction in children with CP can range from potentially life-threatening airway compromise, necessitating intubation or a tracheostomy, to obstructive sleep apnea (OSA) and sleep-disordered breathing (SDB; MacLean et al., 2008, 2012). Sleep-disordered breathing is characterized by intermittent partial or complete airway obstruction with resultant sleep disruption. Sleep-disordered breathing includes OSA, which consists of breathing cessations of at least 10 seconds occurring in the presence of inspiratory efforts during sleep. The reduction of airway size found in children with CP (Imamura et al., 2002) means that they are at increased risk of both SDB and OSA (MacLean et al., 2009, 2012). Polysomnography (PSG) is considered the gold-standard method for diagnosing and determining the severity of OSA and SDB; however, its availability is limited, requires overnight hospital stay, and is expensive to carry out. Consequently, overnight pulse oximetry, which can be carried out at home, has been shown to be a useful tool where PSG is not available (Kaditis et al., 2015).

Obstructive events during sleep can lead to acute and chronic changes in blood pressure and heart rate (Ahmad et al., 2017), with the most severe cases being associated with pulmonary hypertension and cor pulmonale (Maripov et al., 2017; Wong et al., 2017). The SDB can also have a significant deleterious effect on cognition (Montgomery-Downs & Gozal, 2006; O’Brien, 2009), facial development, and weight gain (Pandya & Boorman, 2001), with subsequent “failure to thrive.” There is evidence to suggest that children with CP are at increased risk of impairment in “learning, memory, and cognition” (Broder et al., 1998; Roberts et al., 2012). Studies in infants with Pierre Robin sequence (PRS) have reported an improvement following successful management of SDB, in feeding difficulty, and subsequently weight gain (Lidsky et al., 2008). An observational follow-up study investigating the relationship between SDB in early infancy and outcomes at 3 years of age in children with cleft lip and/or palate has demonstrated that the severity of SDB in infancy had a significant negative impact on neurocognition, quality of life, and weight gain measurable at 3 years of age (Smith et al., 2014).

It is recognized that infant sleep position can affect SDB (Lee et al., 2009; Oksenberg et al., 2010). Guidance on sleep positioning in the United Kingdom recommends back positioning (supine-lying) in infancy to reduce the incidence of sudden infant death syndrome (SIDS; Dwyer & Ponsenby, 1996). However, it is not understood whether this standard sleep positioning advice should be followed by parents of infants with CP. Our survey of practitioners across the United Kingdom demonstrated variability in recommendations given by cleft lip and palate centers (some recommending supine and some side-lying sleep position) and an acknowledgment that further research is needed in this area to determine best practice (Davies et al., 2017). Those cleft centers advising side-lying did so based on clinical experience and perception of improved sleep quality in this position. As a result, there is a gap in evidence about the effectiveness of different sleep positions on SDB in infants with CP. Our feasibility study was undertaken to determine the design of a pragmatic study to investigate the effect of the 2 routinely advised sleep positions in infants with CP on oxygen saturations. This feasibility study included (1) *Pilot home sleep monitoring—*to assess the feasibility of home sleep monitoring (oxygen saturation and ETCO_2_) and to enable sample size calculation for a full trial; (2) *Sleep Questionnaire—*to establish parents perspective of infant’s sleep quality/breathing; and (3) *Qualitative interviews* with parents of infants with CP—to explore parents’ observation of their infant’s sleep, including their awareness of SDB, their experience in taking part in the feasibility study, and their feedback on future trial design (Davies et al., 2018).

## Methods

Four centers, 2 currently advising side-lying sleep positioning and 2 advising supine positioning, were selected to recruit infants born with isolated CP over an 18-month period from (July 2015 to December 2016) to an observational cohort.

All potentially eligible participants were identified by clinical nurse specialists (CNS) and/or consultants at the participating sites. Parents of infants younger than 1 month with isolated CP signed written informed consent to the study following detailed explanation, stressing that there would be no change in the current advice given by individual centers. The protocol was approved by the local research ethics committee (REC Ref: 15/NW/0010).

Infants were excluded if they had any associated syndrome such as PRS, an additional cleft lip, required any immediate intervention to assist breathing (eg, nasopharyngeal airway), any intervention to assist feeding (eg, nasogastric tube), were born preterm (<36 weeks), had known cardiorespiratory disease, or had a family history of SIDS.

Following informed consent, background and demographic information was collected, including the nature of the CP, family history of OSA, smoking habits of family members, and socioeconomic status. Infant weight and length was recorded as standardized deviation score (Cole et al., 1995).

Parents were trained in the recording of blood oxygen saturation (SpO_2_) using pulse oximetry and end-tidal carbon dioxide level (ETCO_2_, as a proxy for partial pressure of CO_2_) using nasal sampling. To limit potential disruption to normal sleep patterns, measurements were undertaken by the parents at home, using a Masimo Radical 7 device (Masimo, California, USA). Monitoring was planned to take place at 1 month (4-7 weeks) and 3 months (10-14 weeks) of age, on at least 2 consecutive sleep periods (days or nights). Parents recorded, in a sleep diary, the starting sleep position and the sleep position when the baby woke. Following each recording, the monitor was collected from the infant’s home and the collected data were downloaded using Stowood Visi-Download software (Stowood Scientific Instruments Ltd, Oxford, UK). Mean SpO_2_, mean SpO_2_ nadir >4%/3%/2%, and mean and median oxygen desaturation index (ODI) -4 and ODI-3 were all recorded from the output and entered into a database. ETCO_2_ was recorded as mean, maximum, and minimum. A working group comprising 5 consultant respiratory pediatricians advised on data analysis and sleep oximetry interpretation. To capture at least 1 sleep cycle, they advised that the minimum length of a saturation study for inclusion in the data analysis should be set at 90 minutes, considered by the respiratory clinicians to be pragmatic time interval that would encompass all the phases of a sleep cycle (MacLean et al., 2015).

Following each recording, parents completed a sleep questionnaire for their infant, to capture information regarding parental perception of sleep quality during the study period. This was adapted from previous validated questionnaires for OSA in children (Brouilette et al., 1984; Chervin et al., 2000) as there was no available validated sleep questionnaire specifically for infants with CP.

Parents were also invited to participate in a qualitative study using telephone or face-to-face interviews (according to parental preference) exploring their understanding of breathing and respiratory effort in infants with CP and their experience of participating in the study. Parents were interviewed after they had either (1) completed 1 or 2 sleep monitoring sessions or (2) declined to participate in sleep monitoring, but consented to be interviewed. Parents participated in semistructured interviews in which the researcher used a topic guide to gather their views of (1) their infant’s sleep behavior, (2) major concerns about sleeping and breathing, (3) sleep positioning, and (4) their experience of participating in the feasibility study and views on a future trial design (specifically randomization). Recruitment continued until theoretical saturation was achieved. Findings regarding participation in this feasibility study and views on a future study are discussed in this article; other detailed results of the qualitative study have been published elsewhere (Davies et al., 2018).

### Outcome Measures

‡ Feasibility—parents’ willingness to participate, likely recruitment and retention, and success and acceptability to parents of domiciliary monitoring of SpO_2_ and ETCO_2_
‡ Primary—level of blood oxygenation saturation measured by pulse oximetry, such as mean SpO_2_, mean SpO_2_ nadir >4%/3%/2%, and mean and median ODI-4 and ODI-3
‡ Secondary—infant’s sleep quality measured by sleep questionnaire completed by their parent

### Sample Size

A total sample size of 30 participants (15 side-lying, 15 supine) was planned as it was deemed adequate to provide preliminary estimates of pulse oximetry measurements at 1 and 3 months after birth in a CP population and to explore differences between supine and side-lying groups. In the event, 17 participants were recruited into side-lying and 13 into supine groups.

All of the clinical results from the feasibility study were reviewed by the study management group in order to make a decision regarding progression to a future larger scale study. All aspects of the feasibility study played a role in the decision-making process.

This report adheres to the CONSORT guidelines for reporting pilot and feasibility clinical trials (Eldridge et al., 2016).

## Results

### Sleep Monitoring

**Figure 1.**
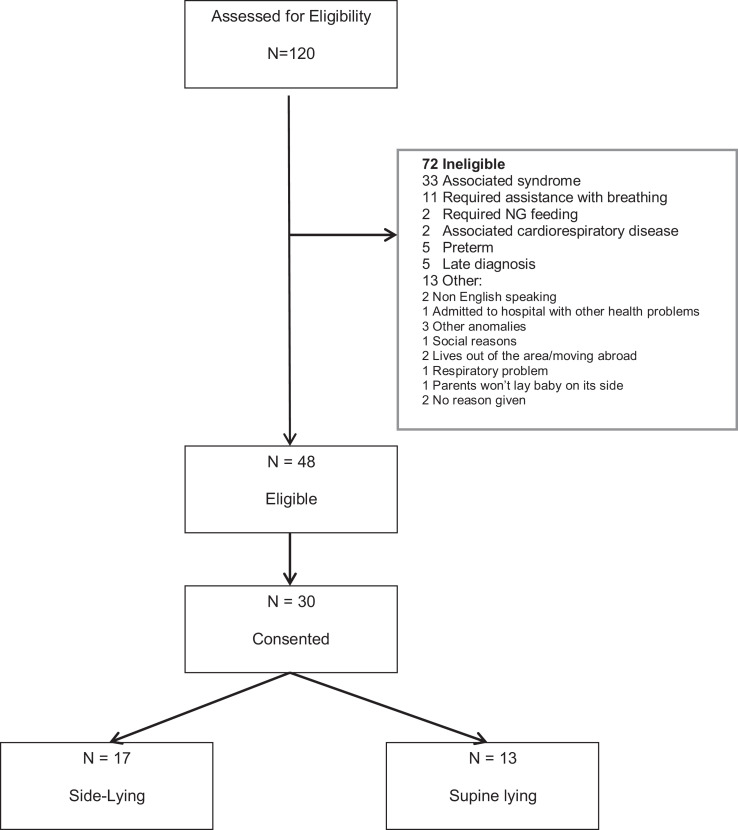
Consort diagram of flow through study (as per CONSORT 2010 statement; Eldridge et al., 2016).

One hundred and twenty infants were assessed for eligibility; 48 were eligible and 30 consented (17 side-lying, 13 supine; Figure 1). Baseline demographic data is provided in Table 1.

**Table 1.** Baseline Characteristics of Infants Side- and Back-Lying.

	Side-lying (Manchester and Liverpool sites)	Supine-lying (Leeds and Newcastle sites)
Total	n = 17	n = 13
Male gender; n (%)	8 (47.1%)	7 (53.8%)
Age in days, mean (SD) At time of consent	32.8 (11.5)	35.4 (15.3)
Birth weight (kg)	(n = 17)	(n = 12)
Mean (SD)	3.30 (0.56)	3.37 (0.48)
Mean *z* score	−0.19	−0.15
Weight at recruitment (kg)	(n = 17)	(n = 11)
Mean (SD)	3.88 (0.79)	3.94 (0.53)
Mean *z* score	−1.02	−1.14
Smokers in the household; n (%)	4 (23.5%)	5 (38.5%)

At 1 month of age, 13 side-lying babies provided oxygen saturation monitoring data over 24 separate sleep sessions (13 sessions >90 minutes in length) and 11 supine-lying babies provided data over 26 sleep sessions (21 sessions >90 minutes in length). At 3 months of age, 10 side-lying babies provided oxygen saturation monitoring data over 24 sleep sessions (12 sessions >90 minutes in length) and 9 supine-lying babies provided data over 26 sleep sessions (15 sessions >90 minutes in length). The median length of recordings used in the analysis at 1 month was 2 hours 36 minutes and 3 hours 18 minutes and at 3 months was 4 hours 19 minutes and 3 hours 38 minutes (for side-lying and back-lying, respectively). Summary statistics for sleep sessions >90 minutes are provided in Table 2. Mean SpO_2_, mean nadir >4% /3% /2%, and median ODI-4 and ODI-3 were similar between side- and supine-lying babies at both time points. Mean ODI-4 and ODI-3 was lower for side-lying infants at 1 and 3 months. On reviewing the individual data, there appeared to be considerably more variability in the oxygen saturation measurements of the supine-lying infants.

**Table 2.** Data From Saturation Recordings >90 Minutes Long at 1 and 3 Months in Side- and Supine-Lying Babies.

	Side-lying (Manchester and Liverpool sites)	Supine-lying (Leeds and Newcastle sites)	Side-lying (Manchester and Liverpool sites)	Supine-lying (Leeds and Newcastle sites)
	@ 1 month	@ 1 month	*@ 3 month*	@ 3 month
Number of sleep sessions >90 minutes	13	21	*12*	15
Median (IQR) number of sleep session/baby	2 (2-2)	2 (2-3)	2 (2-2)	2 (2-3)
Median length of sleep study	2 hours 36 minutes	3 hours 18 minutes	4 hours 19 minutes	3 hours 38 minutes
Mean SpO_2_				
Mean (SD)	97.78 (1.46)	97.76 (1.69)	*97.99 (1.77)*	97.94 (1.58)
Minimum	95.13	92.49	*94.00*	93.7
Maximum	99.58	99.57	*99.43*	99.52
Median (IQR)	97.72 (97.17-99.08)	98.13 (97.21-98.64)	*98.7 (97.51-99.14)*	97.85 (97.34-99.34)
Mean nadir >4%				
Mean (SD)	90.79 (2.26)	91.13 (2.10)	*90.67 (2.61)*	90.68 (1.40)
Minimum	85.33	86.32	*84.91*	88.15
Maximum	93.67	94.50	*94.50*	92.3
Median (IQR)	91.32 (90.56-91.76)	91.70 (90.63-92.39)	*90.88 (89.30-92.73)*	91.32 (89.61-91.61)
Mean nadir >3%				
Mean (SD)	91.91 (2.17)	92.33 (1.96)	*91.87 (2.53)*	91.78 (1.50)
Minimum	86.40	87.46	*85.81*	89.06
Maximum	94.24	95.62	*94.86*	93.52
Median (IQR)	92.59 (91.64-93.40)	92.73 (91.81-93.53)	*92.47 (90.57-93.79)*	92.17 (91.07-92.81)
Mean nadir >2%				
Mean (SD)	93.26 (1.81)	93.62 (1.93)	*93.42 (2.41)*	93.23 (1.47)
Minimum	88.64	89.00	*86.94*	90.57
Maximum	95.48	95.69	*95.85*	94.90
Median (IQR)	93.81 (92.64-94.23)	94.00 (93.52-94.88)	*93.62 (92.98-95.08)*	93.47 (92.56-94.27)
ODI Dips/ Hr >4%				
Mean (SD)	17.52 (14.28)	28.21 (28.67)	*11.66 (9.31)*	26.4 (39.70)
Minimum	1.89	2.28	*1.4*	1.64
Maximum	59.05	92.39	*35.7*	144.37
Median (IQR)	15.91 (10.37-20.17)	17.57 (7.45-39.12)	*10.69 (5.51-15.44)*	9.78 (5.75-17.34)
ODI Dips/ Hr >3%				
Mean (SD)	26.32 (19.06)	36.28 (30.64)	*16.19 (11.54)*	37.17 (45.62)
Minimum	3.16	2.86	*3.03*	6.56
Maximum	75.84	100.50	*43.57*	162.25
Median (IQR)	25.73 (12.59-36.36)	25.02 (11.69-54.77)	*15.99 (6.85-21.74)*	14.76 (7.49-75.66)

Abbreviation: ODI, oxygen desaturation index.

The majority of parents were unable to carry out the ETCO_2_ monitoring as they found it disturbed their babies and results on only 12 infants at visit 1 and 6 at visit 2 were received, only 1 of which was >90 minutes.

### Sleep Questionnaires

Twenty-nine (17 side-lying, 12 supine) parents completed the sleep questionnaire at 1 month of age. Although only 3 (10%) parents reported that their baby did not have good quality sleep, 8 (28%) went on to describe their baby’s sleep as poor/restless or sometimes restless (3 every day; 2 >3 d/wk; 3 ≤3 d/wk). In addition, 38% (47% side-lying, 25% supine) reported that their child had difficulty breathing while asleep at some time (1 every day; 3 >3 d/wk; 3 ≤3 d/wk; 2 every 1-2 weeks; and 2 only when they had a cold). Ten percent (3 parents) reported that their child had stopped breathing for periods or had pauses in their breathing during their sleep at times (1 everyday; 1 >3 d/wk; 1 ≤3 d/wk; 1 side-lying, 2 supine).

Three-quarters (76%) of parents described their baby as snoring or noisy when sleeping (12 every day; 4 >3 d/wk; 3 ≤3 d/wk; 2 every 1-2 weeks; and 1 only when they had a cold; 94% of side-lying, 50% of supine). In addition, a third of parents also described their baby snoring or making snoring noises when awake (2 every day; 5 >3 d/wk; 1 ≤3 d/wk; 2 every 1-2 weeks).

Four parents, whose babies slept on their side, reported having to reposition their babies on to their side to improve their sleep quality, perhaps implying that they did not always stay in the original position they had been placed, although this was not specifically stated by parents.

At 3 months, 22 parents (11 side-lying, 11 supine) completed the sleep questionnaires. Nine parents still reported their infant had difficulty breathing when asleep, but 5 of these were only when they had a cold. In addition, 4 parents reported that their child had stopped breathing for periods or had pauses in their breathing during their sleep at times (1 everyday; 1 ≤3 d/wk; 2 every 1-2 weeks; 3 side-lying, 1 supine). Noisy or snoring breathing, both during the day (41% of parents reported this) and at night time (86% of parents) remained very common.

At 3 months, 3 parents regularly repositioned their baby during sleep; all reported they moved them onto their side as they slept easier that way (including 1 supine-lying baby).

### Qualitative Interviews

Parents of 27 infants with CP were interviewed. Parents reported observing their babies during sleep to ensure they were breathing. Although they described signs such as snoring, they showed little awareness of SDB and its potential long-term consequences. Parental decision to use side-lying or supine sleep positioning reflected their response to advice from CNS, observation of their infant’s comfort, ease of breathing, and experience of infant care.

All parents indicated strong interest to participate in further studies evaluating the effects of sleep position. Less than half said they would be reluctant to participate in a study that involves randomization to sleep position. The acceptance of randomization by the remaining half was qualified because they would not comply if they thought the baby was uncomfortable in the allocated position. Parents’ willingness to be randomized was not guided by the sleep position used in the current feasibility study.

Parents’ main concern about taking part in a future study was the use of nasal cannula to monitor ETCO_2_, which many parents felt caused distress in their infant and sometimes led to the discontinuation of monitoring. In a few cases, parents withdrew their participation due to the perceived distress of their infants caused by the nasal cannula. Parents reported that the information provided for home monitoring was clear and concise, but sometimes found the saturation/ETCO_2_ machine intimidating. These parents were uncomfortable about the “clinical” appearance of the equipment when used in the home. They also reported concern that they or young children in the home could break the machine. Parents described the factors that could affect the length of monitoring including parents’ confidence with using the equipment, how settled the infants were, and disruptions due to domestic circumstances.

## Discussion

This feasibility study was aimed at gathering the information necessary to design and conduct a future study to establish the best sleeping position for infants with CP. We have observed that this study design is feasible for a larger study with minor modifications, and this unanswered question is of importance to parents and clinicians.

Clinical practice in the United Kingdom is inconsistent in the advice about sleep position given to families with infants with CP. All parents agreed that it was important to have evidence supporting advice regarding safe sleep, but almost half of the interviewed parents expressed strong reluctance to participate in randomization of sleep positioning; while others indicated they would agree to be randomized to one of the 2 sleep positions but might change the position if they perceive the infant to be uncomfortable (Davies et al., 2018). As such, we believe a comprehensive cohort study, comprising randomized and nonrandomized arms, would maximize the number of parents able to participate and the scientific value of the study. Consequently, infants meeting the inclusion criteria and whose parents are willing for their child to take part, but not to be randomized, will be included as part of the cohort study (parents asked to adhere to the standard advice given by their cleft center). Parents who consent for their infant to be randomized will be included in the randomized control trial (parents asked to adhere to the randomly allocated sleep position). Informed by parents and cleft nurse specialists, we believe this study design will maximize recruitment and retention. Infants in the cohort study will follow an identical protocol, except for not being randomized to sleep position.

Most parents disliked the nasal canulae that measured the exhaled CO_2_ (ETCO_2_) because they thought it disturbed and/or distressed their infant. Only 1 infant completed recordings ≥90 minutes. Feedback from several recruiting nurses via the Study Advisory Group was that many parents failed to carry out any of the recordings including the saturation monitoring because they disliked the experience of trying to carry out the ETCO_2_ monitoring. Hence, it would not be feasible to include this in the definitive study. Although this is disappointing, as it has been suggested that in infants with minor degrees of intermittent airway obstruction the first change may be not a fall in blood oxygenation but a rise in expired CO2 or a rise in respiratory rate, these minor episodes will likely be missed in both side- and back-lying groups and not affect the overall outcome of the trial (D’Souza et al., 2020).

Home oxygen saturation monitoring was much more acceptable to parents than ETCO_2_ recording and was completed in at least 1 time point in all but 3 babies. Duration of recording was inadequate for inclusion in the data analysis in a large proportion of the babies. Perhaps, it was reflecting the pattern of sleep in this age group (ie, frequent short sleeps interspersed with feeds). It is possible, this pattern of sleep may be related to CP, as a study of healthy infants of a similar age has demonstrated that between 88% and 92% of infants managed SpO_2_ recordings of >4 hours duration, compared with 55% to 70% infants with recordings >90 minutes in this study (Evans et al., 2018). However, it was notable that as researchers became more familiar with the study over time they encouraged parents to carry out the recording at a time when the baby was most likely to have a longer sleep (ie, night time), and this resulted in a higher success rate. In addition, as the study progressed, parents appeared to have a better success rate with the length of recording. Possibly, this was in part due to the researchers giving better explanations to parents as to what was needed and how to carry out the recordings. Of note, we did not tell parents that recordings needed to be at least 90 minutes in duration and this might have resulted in recordings being cut short unknowingly. For the future study, we have suggested that parents only record overnight that they record for at least 5 hours and document if the child wakes or feeds during that time and that the recording be done on 2 separate nights to try and maximize sleep recording time.

Despite clear and concise instructions for the study equipment, some parents expressed concerns about the complexity of the equipment and researchers suggested, based on parents’ feedback, the use of an instruction video would aid any future study. This should provide information whenever and wherever parents need it. Parents also indicated some reasons why the monitoring was sometimes short including how settled the baby was, other domestic circumstances, and confidence using the equipment (Davies et al., 2018), which are important to address in the future trial. Some parents suggested that if they were allowed more time with the machine and equipment in the future study, they might be able to make more attempts in recording. This would, subsequently, increase their confidence in operating the machine and overcome unforeseeable domestic circumstances.

The SpO_2_ results from this study suggest that there was greater variability in the supine-lying group. We observed that the mean 4% ODI values for infants in the side-lying cohort approximated to values reported for healthy infants of the same age in a recent cohort study of healthy, normally developing infants (non-CP; Evans et al., 2018), whereas the mean 4% ODI values for the back-lying cohort were markedly higher. Oxygen desaturation index (ODI) is the number of times per hour of sleep that the blood oxygen level drops by a certain degree from baseline; it is recognized as a marker for OSA (Kaditis et al., 2016). We believe the variability in 4% ODI we have demonstrated in this small number of CP infants justifies pursuing this practical methodology for our future proposed large study, recognizing that it is not the gold-standard test for SDB.

The important baseline characteristics of the side- and supine-lying cohorts were similar, which gives confidence to the study size calculation for a definitive trial. We have gathered valuable information regarding parents preferences, for example, ETCO_2_ monitoring, which we will implement in a future trial design to maximize patient retention and quality of the collected data.

Study limitations included short observed sleep cycles as compared to the studies published in other infant groups (Evans et al., 2018). However, despite that we were able to capture a full sleep cycle (90 minutes) in 60% of recordings. Many children were able to provide measurements for at least 1 monitoring period. A limitation of this study is the lack of PSG for comparison with the oximetry readings. This study was deliberately designed with a pragmatic approach in order to limit cost, impact on families, and to mirror current practice particularly in resource-limited settings where the access to PSG is restricted. We recognize that oximetry is likely to underestimate OSA in our population, but a positive finding in such a study will highlight the need for further investigation into sleep position in this cohort. It is of note that parents were not keen to carry out ETCO_2_ monitoring as it was perceived as distressing to their baby, one could extrapolate that these parents would also be unwilling for an inpatient stay for a full PSG for similar reasons.

This feasibility study provides the necessary information to enable the design of a future pragmatic study to enable us to develop a better understanding of the importance of sleep position and SDB for infants with CP. Optimizing and standardizing the recommended sleep position for infants with CP has clear potential health advantages, not least in terms of facial development, cognitive functioning, weight gain, and the avoidance of significant cardiorespiratory complications. Clinical nurse specialists support a future study to enable them to provide guidance to parents based on an evidence base (Davies et al., 2017). The qualitative findings about parents’ perspectives on SDB and sleep provide practitioners with information about the concerns and perspectives of parents, highlighting the need for better patient information to explain SDB and its potential sequelae.

Health care professionals face a clinical dilemma between adhering to standard “back to sleep” guidance and responding to clinical assessment of respiratory effort for infants with CP. In the absence of clear evidence, specialist centers rely on clinical judgment regarding respiratory problems to identify what they believe is the most appropriate sleeping position for infants with CP. Clearly, that advice continues to be different in different centers across the country. Further research is needed to determine the best sleep position for an infant with CP. Based on the findings from this feasibility study, we propose a comprehensive pragmatic cohort study incorporating a parallel group randomized controlled trial of side-lying compared with supine sleep positioning in infants with CP and would represent an important step toward a better understanding of SDB in this patient group.

## Appendix

On behalf of the SLUMBRS Study Advisory Group (SAG) and Respiratory Group: H. Robson (Cleft Palate CNS, Manchester), R. Sammon (Patient representative), N. Hudson (Cleft Palate CNS), W. Shaw (Orthodontist, Manchester), C. Couhig (Cleft Palate CNS, Newcastle), R. Mattick (Consultant Orthodontist, Newcastle), D. Beaumont (Cleft Palate CNS, Leeds), E. Blair (Cleft Palate CNS, Leeds), S. Wilkinson (Respiratory Paediatrician, Manchester), W. Carroll (Respiratory Paediatrician, Derby), H. Elphick (Respiratory Paediatrician, Sheffield), L Turnbull (Respiratory Paediatrician, Manchester), N. Mercer (Consultant Plastic Surgeon, Birmingham), D. Wynne (Consultant ENT Surgeon, Glasgow), D. Stokes (Chief Executive, CLAPA), N. Harman (Research Associate Biostatistics, Liverpool), C. Bennett (Healing Foundation Cleft and Craniofacial Clinical Research Centre, Manchester), C. Wright (BRC, Manchester), N. Brown (Research Coordinator, Manchester)
